# Level of and associated factors for non-adherence to anti-tuberculosis treatment among tuberculosis patients in Gamo Gofa zone, southern Ethiopia: cross-sectional study

**DOI:** 10.1186/s12889-020-09827-7

**Published:** 2020-11-13

**Authors:** Dessalegn Ajema, Tamiru Shibru, Temesgen Endalew, Selamawit Gebeyehu

**Affiliations:** 1 School of Public Health, College of Medicine and Health Sciences, Arba Minch University, Arba Minch, Ethiopia; 2 School of Medicine, College of Medicine and Health Sciences, Arba Minch University, Arba Minch, Ethiopia

**Keywords:** TB, Non-adherence, Gamo Gofa, Ethiopia

## Abstract

**Background:**

Non-adherence to anti-TB treatment is one of the crucial challenges in improving tuberculosis (TB) treatment outcomes and reducing healthcare costs. The poor adherence to anti-TB treatment among patients with TB is a major problem in Ethiopia. This study aimed to assess the level of and associated factors for non-adherence to anti-TB therapy among patients with tuberculosis in the Gamo Gofa Zone.

**Methods:**

A cross-sectional study was conducted at Gamo Gofa Zone from July 20 – August 30, 2017. A multi-stage sampling technique was used. The study included 289 patients who were on anti-TB treatment. Data were collected by trained data collectors using a structured and pre-tested questionnaire through interviews. A multiple logistic regression model was fitted using SPSS 23 to identify factors associated with non-adherence to anti-TB treatment at a 5% significance level.

**Results:**

We found that 16.5% of the participants were non-adherent for anti-TB treatment. Failure to disclose one’s TB status to his or her family (AOR = 31.7; 95% CI: 9.1–111.1), having no information on the expected adverse events (AOR = 31.1; 95% CI: 7.5–128.3), past anti-TB treatment history (AOR = 5.3; 95% CI: 1.5–18.8) and a smoking cigarette (AOR = 11.7; 95% CI: 3.2–43.03) were found to be associated with a higher odds of being non-adherent to anti-TB treatment.

**Conclusions:**

The level of non-adherence to anti-TB treatment among TB patients was high. Health care providers should counsel TB patients on the expected adverse events and measures to be taken when patients face the expected adverse events. They should also counsel their patients to disclose their TB status to his or her family and for ceasing cigarette smoking.

**Supplementary Information:**

The online version contains supplementary material available at 10.1186/s12889-020-09827-7.

## Background

Tuberculosis (TB) is an infectious disease caused by the bacillus *Mycobacterium tuberculosis*. It typically affects the lungs (pulmonary TB), but can also affect other sites (extrapulmonary TB) [1]. Most people who had TB disease can be cured with early diagnosis and correct treatment. Despite this, it has been continued as a leading cause of death from a single infectious agent ranking on top of HIV/AIDS for the past five years [2].

In 2018, TB caused an estimated 1.2 million deaths (range, 1.1–1.3 million) among HIV-negative people and 10.0 million people developed the disease. The biggest burden (83%) of TB deaths among HIV-negative people shouldered by the African and South-East Asia Region. Ethiopia is in the list of 30 high burden countries (HBCs), with an estimated total incidence of TB 165 per 100,000 population [3].

Based on the evidence that TB is a global public health challenge, WHO launched different strategies like the Directly Observed Treatment, Short-course (DOTS), STOP TB Strategy and End TB strategy for TB control [1–5]. Almost all countries, including Ethiopia, have adopted this strategy and have been implementing for years. Similarly, in recent years, they committed and have targeted to end the epidemics of AIDS, TB, and other communicable diseases by 2030 [2].

Even though many national and international efforts have been implemented against TB prevention and control, currently, the world as a whole, are not on track to reach the 2020 milestones of the End TB Strategy [3]. Besides this, drug-resistant TB threatens global TB care and prevention and remains a major public health concern in many countries [2]. The year 2019 WHO report shows in 2018, there were about half a million new cases of rifampicin-resistant TB (of which 78% had multidrug-resistant TB (MDR-)TB, and many cases are developing Extensively Drug resistance (XDR) TB among retreatment cases throughout the world [3].

Drug-resistant (DR-) TB emerges at least in part due to inappropriate treatment or suboptimal adherence to the treatment regimen [6]. Thus poor adherence to a prescribed treatment increases the risks of morbidity, mortality, and drug resistance [7]. As a result, patients with TB are expected to have non-adherence levels of less than 10% to facilitate cure [8, 9].

Non-adherence rates to long-term medication including TB and HIV/AIDS patients in developing countries seem to be higher and remain a challenge [7, 10]. In sub-Saharan Africa, there is a high rate of losses to follow-up of TB patients that range from 11.3 to 29.6% [11]. Mapping of TB treatment outcomes in Ethiopia stated that 0.7% of the patients had treatment failure, and 5.5% were lost to follow-up. The overall prevalence of poor TB treatment outcomes was 9.0%, which is most probably due to treatment non-adherence and loss to follow up [12]. A systematic review done to estimate the pooled prevalence of non-adherence to anti TB treatment in Ethiopia showed Southern Nations and Nationalities of Ethiopia had the highest (23.61%) pooled prevalence estimate of non-adherence to anti TB treatment [13].

To improve TB treatment outcomes, it requires a better understanding of factors that influence TB treatment non-adherence. Studies conducted in different parts of Ethiopia revealed several factors associated with anti-TB treatment non-adherence, including factors related to the health system, patient and socio-economic characteristics, [14–16]. As TB treatment non-adherence is an “unavoidable by-product of collisions between the clinical world and the other competing worlds of work, play, friendships and family life” the factors affecting it can be changed over time [7]. The present study was therefore conducted to assess the level and factors associated with non-adherence to anti-TB treatment among TB patients in Gamo Gofa Zone, Ethiopia.

## Methods

### Study setting

The study was conducted in the Gamo Gofa Zone of Southern Nations, Nationalities and Peoples` Regional State (SNNPR), Ethiopia. It is found at 5^0^ 57–6^0^ 71′ N 36^0^ 37′- 37^0^98′ E and altitude of 680 to 4207 masl. The general elevation of the Gamo Gofa zone ranges from 680 to 4207 m above sea level. Gamo Gofa has a population of about 2,040,972. DOTS services have been provided for years in the area. Seventeen health facilities within this zone register and treat all diagnosed TB patients under the DOTS. In this facility’s diagnosis, treatment, and prevention services have been given. Health extension workers play an important role in the identification of presumptive cases at the community level. Once a presumptive case is identified they immediately refer to a health facility providing DOTS service for diagnosis and treatment. In line with the national TB and leprosy control program guidelines [17], once diagnosis is made, patients are provided with anti-TB medication and care free of charge. According to the zonal report, TB morbidity is decreased from 41% in 2017 to 37% in 2019, while the mortality also decreased from 2.4% in 2017 to 2.3% in 2019. Moreover, there were 3.2% treatment failure cases in 2017 and 1.2% cases in 2019. The report also stated that the drug-resistant (DR) TB rate was 3.4% in 2017 increased to 4.2% in 2019 among new cases and 12.5% in 2017 increased to 15.2% in 2019 among previously treated cases. The study was conducted from July 20, 2017 – August 30, 2017.

### Study design and population

A health facility-based cross-sectional study was conducted. All patients aged over 15 years, who had started anti- TB treatment and on treatment follow-up in selected health facilities were eligible.

### Sample size and sampling procedures

To reach the participants, a Multi-stage sampling method was used. From 17 woreda health offices and one general hospital, by using a simple random sampling method four of the registry centers were selected. Proportionate allocation to size was made. Finally, the study participants were selected by simple random sampling from the sampling frame (patients’ register). The sample size was calculated using a single population proportion formula; considering 13.6% level of non-adherence [15] with a 5% margin of error and 95% confidence, it was 183. The design effect was taken into consideration and the sample size was multiplied by 1.5, giving 275. By adding a 5% none response rate the final sample size was 289.

### Data collection tool

Data were collected using an interviewer-administered questionnaire. The questionnaire was developed based on the comparable studies [6, 14–16, 18–20] and included demographics (age and sex), economic status (monthly income), social (residence, level of education, employment, religious status, ethnicity, and marital status), medical history(number of doses taken in the month before the study interview, history of previous anti-TB treatment and history of opportunistic infection), TB knowledge (etiology, mode of transmission, symptoms, treatment duration and effectiveness, and expected adverse events), health provider (counseling about the expected adverse effect), and health system (time to reach a health facility, waiting time and mode of transportation) and patients behaivor (smoking, alcohol drinking, khat chewing and disclosing one’s TB status),(See additional file questionnaire for the study). It was initially prepared in English and then translated into Amharic and back-translated to English to ensure its consistency. The questionnaire was pretested before the actual data collection. Data was collected through a face-to-face exit interview. It was collected after verbal informed consent was obtained. Interviewers were specifically trained for this study.

### Operational definitions

Non-adherence for TB treatment: If the patient had taken less than 90% of the dose prescribed within a month he/she was regarded as non-adherent [5, 10].

### Data analysis

Data were coded and entered into Epi Data version 3.1 [21] and exported to SPSS version 23 [22] for analysis. The participant’s characteristics were summarized using frequencies and percentages for categorical variables. Furthermore, patients who had the correct answer to three questions was taken as having good knowledge while the incorrect answer to three or more question was taken as having poor knowledge. Bivariate logistic regression analysis was conducted to assess the association between each categorical variable and the non-adherence to anti-TB treatment. The variables with *p*-value ≤0.25 were included in the multivariable logistic regression model. The multiple logistic regression model was fitted using a backward stepwise selection method to identify variables independently associated with the outcome variable of interest at a 5% significance level. The final model was assessed using the Hosmer and Lemeshow goodness of fit test. The adjusted odds ratios (aORs) and the corresponding 95% confidence intervals (CIs) of the variables in the final model were reported.

### Ethical consideration

Ethical clearance was obtained from Arba Minch University research and community service core process ethical review board, and permission was secured from the Gamo Gofa zone health Department. Participants were informed about the objectives, risks, and benefits of the study. Verbal consent was obtained for those study participants who were18 years and above. Verbal Assent was obtained for participants less than 18 years from themselves and if families were available parental permission was asked; if parents were not available permission was taken from the health facility or health service provider. Participant’s involvement in the study was voluntary and those who wish to quit their participation at any stage were informed to do so without any restriction.

## Results

A total of 249 participants were included in this study with a response rate of 86.2%. One hundred fifty-five (62.2%) of study participants were rural residents and 148 (59.4%) were males. One hundred twenty-three (50.4%) of them did not attend any formal education and 77 (30.9%) of the respondents were farmers. One hundred forty-eight (59.4%) were protestants and 194 (77.9%) participants were Gamo in Ethnicity (Table 1). One hundred eighty-nine (75.9%) of the participants walk to the health facility. For one hundred twenty-five (50.2%) participants, it took them more than 30 min to reach a health facility.

**Table 1:** Socio-demographic and economic characteristics of participants who started anti-tuberculosis treatment and are on follow up in health institutions of Gamo Gofa Zone, 2017 (*n* = 249)

Variable		Number	Percent (%)
Residence	Urban	94	37.8
	Rural	155	62.2
sex of respondent	Male	148	59.4
	Female	101	40.6
Age of respondent (years)	15–24	80	32.1
	25–34	72	28.9
	35–44	44	17.7
	≥45	53	24.3
Marital status	Married	149	59.8
	Single	82	32.9
	Divorced/Widowed	18	7.2
Level of education	Unable to read and write	87	34.9
	Read & write	36	14.5
	grade1–8	57	22.9
	grade 9–12	43	17.3
	College and above	26	10.4
Religion	Orthodox	84	33.7
	protestant	148	59.4
	Muslim	17	6.8
Ethnicity	Gamo	194	77.9
	Gofa	16	6.4
	other	39	15.6
occupation	Housewife	42	16.9
	Daily laborer	46	18.5
	Government employee	19	7.6
	farmer	77	30.9
	Private employee	20	8.0
	Other (specify)	45	18.1
Income (in birr)	< 500	91	36.8
	≥500	158	63.5

The overall level of non-adherence to anti-TB treatment over one month and the last four days before the survey were 16.5 and 10%, respectively. One hundred sixty-six (66.7%) participants had good knowledge about TB. From all participants, 185 (74.3%) of them had a family member or friend who reminds the patient to take treatment, and 37(13.4%) smoke cigarettes; the adverse event was experienced by 102(41.0%) participants. From different adverse events, urine discoloration was experienced by 56(54.9%) of them. Symptoms of TB were reported by 36(14.5%) of the participants at the time of the interview. One hundred eighty-two (73.1%) of them acquired Pulmonary TB confirmed with sputum and 36(14.5%) of them had pulmonary TB without sputum. Extra-pulmonary TB (EPTB) was found among 31 (12.4%) of the participants. From all participants, 198 (79.5%) had no history of previous anti-TB treatment. Nearly all of them 248(99.6%) had screened for HIV and 14(5.6%) of them were positive for HIV. Those participants who were positive for HIV had started Anti-Retroviral Treatment (ART) and had no other Opportunistic Infections (OIs) (Table 2).

**Table 2:** Determinants of anti-TB treatment non-adherence among participants who started anti-tuberculosis treatment and are on follow up in health institutions of Gamo Gofa Zone, 2017

Variable		number	percent
Family member or friend who reminds the patient to take treatment	yes	185	74.3
	no	64	25.7
Knowledge about TB	good	166	66.7
	poor	83	33.3
TB status disclosure to the family	disclose	211	84.7
	not disclose	38	15.3
Felt discriminated by family/Community	Discriminated	23	9.2
	Non discriminated	226	90.8
Support for medication	health professional	26	10.4
	family member	185	74.3
	No treatment supporter	38	15.3
Chewing khat	yes	27	10.8
	no	222	89.2
Addicted to alcohol	yes	43	17.3
	no	206	82.7
Smoking	yes	34	13.7
	no	215	86.3

Income, marital status, time to reach a health facility, waiting time to get served, information on the expected adverse event, missing drug in the last four day, alert for medication, knowledge about the disease, disclose one’s TB status, discrimination from family and society, use of alcohol, Khat, smoking cigarette, having adverse event t and past anti-TB treatment history had a *p*-value of less than 0.25 in the bivariate analysis. Thus, these variables were considered for the multivariable logistic regression analysis. In the multivariable analysis, information on the expected adverse event, having past anti-TB treatment history, disclose one’s TB status to family, and smoking cigarettes had a statistically significant association with the level of non-adherence to anti-TB treatment (Table 3).

**Table 3:** The result of Bivariate and multivariable logistic regression for non-adherence among participants on anti-tuberculosis treatment and on follow up in health institutions of Gamo Gofa Zone,2017

Variable	non-adherence		COR(95%CI)	AOR(95%CI)
	Yes *n* (%)	No *n* (%)		
Sex				
**Male**	24 (16.2)	124 (83.8)	1	
**Female**	17 (16.8	84 (83.2)	1.05 (0.53–2.06)	
Age				
**15–24**	13 (16.3)	67 (83.8)	1	
**25–34**	14 (19.4)	58 (80.6)	1.24 (0.54–2.86)	
**35–44**	9 (20.5)	35 (79.5)	1.33 (0.52–3.4)	
**≥ 45**	5 (9.4)	48 (90.6)	0.54 (0.179–1.61)	
Income				
**< 500**	21 (23.1)	70 (76.9)	2.07 (1.05–4.072)^a^	1
**≥ 500**	20 (12.6)	138 (87.3)	1	1.7 (0.4–7.6)
Marital status				
**married**	14 (9.3)	135 (90.6)	1	1
**single**	20 (24.4)	62 (75.6)	3.11 (1.47–6.56)^a^	5.1 (0.98–26.4)
**divorced**	7 (38.9)	11 (61.1)	6.14 (2.05–18.35)^a^	23.8 (2.4–231.1)
Mode of transportation				
**waking**	27 (14.3)	162 (85.7)	0.55 (0.27–1.13)	
**public**	14 (23.3)	46 (76.7)	1	
Time to reach a health facility				
**≤ 30 min**	12 (9.6)	112 (90.3)	1	1
**> 30 min**	29 (23.2)	96 (76.8)	2.82 (1.36–5.83)^a^	1.4 (0.3–2.7)
Waiting time				
**≤ 30 min**	20 (9.4)	192 (90.6)	1	1
**> 30 min**	21 (56.7)	16 (43.2)	12.6 (5.68–27.66)^a^	5.2 (0.8–30.4)
**Have you consulted**				
**Yes**	40 (16.5)	203 (83.5)	1	
**No**	1	5	1.01 (0.12–8.92)	
Information on the expected adverse event				
**Yes**	21 (9.4)	202 (90.6)	1	1
**No**	20 (76.9)	6 (23.1)	32.06 (11.6–88.7)^a^	**31.1 (7.5–128.3)**^**b**^
Knowledge				
**Good**	13 (7.8)	153 (92.2)	1	1
**Poor**	28 (33.7)	55 (66.3)	5.99 (2.89–12.38)^a^	5.5 (0.94–30.7)
**use traditional medicine**				
**yes**	11 (23.9)	35 (76)	1.81 (0.83–3.95)	
**No**	30 (14.8)	173 (85.2)	1	
Alert				
**Yes**	16 (8.6)	169 (91.4)	1	1
**No**	25 (39.1)	39 (60.9)	6.77 (3.3–13.87)^a^	2.6 (0.43–15.1)
Disclosing one’s TB status				
**Yes**	15 (7.1)	196 (92.8)	1	1
**No**	26 (40)	39 (60)	28.3 (11.95–67.05)^a^	**31.7(9.1–111.1)**
Discriminated				
**Yes**	9 (39.1)	14 (60.8)	3.89 (1.56–9.75)^a^	5.9 (0.7–48.6)
**No**	32 (14.2)	194 (85.8)	1	1
Addicted to alcohol				
**Yes**	16 (37.2)	27 (62.8)	4.29 (2.03–9.05)^a^	0.6 (0.1–3.95)
**No**	25 (12.1)	181 (87.8)	1	1
Addicted to khat				
**Yes**	18 (66.7)	9 (3.3)	17.3 (6.97–42.95)^a^	2.03 (0.02–18.05)
**No**	23 (10.4)	199 (89.6)	1	1
Smoke cigarette				
**Yes**	24 (70.6)	10 (29.4)	27.9 (11.5–67.9)^a^	**11.7 (3.2–43.03)**^**b**^
**No**	17 (7.9)	198 (92.1)	1	1
Adverse event				
**Yes**	23 (22.5)	79 (77.5)	2.08 (1.06–4.108)^a^	1.3 (0.3–6.4)
**No**	18 (12.2)	129 (87.7)	1	1
Previous TB treatment				
**Yes**	16 (31.4)	35 (68.6)	3.16 (1.53–4.6.53)^a^	**5.3(1.5–18.8)**^**b**^
**No**	25 (12.6)	173 (87.4)	1	1
Having a symptom of TB				
**Yes**	10 (27.9)	26 (72.2)	2.3 (0.99–5.14)	
**No**	31 (14.6)	182 (85.4)	1	

^a^ *P*-value≤0.25; ^**b**^ *P*-value< 0.05

The odds of TB treatment non-adherence among the study participants who didn’t disclose their TB status to their families was higher compared to those who did disclose their TB status told (aOR = 31.7; 95% CI: **9.1–111.1).** The study participants who had no information on the expected adverse events had higher odds of the TB treatment non-adherence compared to those who had information (aOR = 31.1;95% CI:**7.5–128.3).** Furthermore**,** the non-adherence to TB treatment was higher among those with TB treatment in the past (aOR = 5.3; 95% CI: **1.5–18.8)** and those who smoke a cigarette (aOR = 11.7; 95% CI: **3.2–43.03).**

## Discussion

In the present study, 16.5% were non-adherent to anti-TB therapy in the last month before the interview. This finding was higher than the levels of non-adherence reported from another study in Northwest Ethiopia (10%) [15]. This variation might be from differences in participants. Such that, in this study, we included both new TB cases and patients who had past treatment but the study in Northwest Ethiopia included only new TB cases. So, patients who had past treatment might be reluctant or unwilling to try it again because of the previous experience which could be treatment failure. The level of non-adherence among new TB cases in this study was 10% which is similar to the report from the study in Northwest, Ethiopia [15].

However, the present non-adherence level over one month was lower than the previous reports from Southern Ethiopia (24.7%), Mekelle (55.8%), and Uganda (25%) [14, 16, 18]. This is probably due to variation in the participant characteristics and duration in which adherence was measured. For example, in this study, we included both HIV-positive and HIV-negative patients but the study in Mekelle, Uganda, included only TB/HIV co-infected patients in which HIV co-infected patients are more likely to be non-adherent because they have to get HIV care in addition to the TB care [8]. Besides, this variation may be attributable to the duration in which the adherence was calculated. In the case of the Ugandan study, adherence was estimated over the last five days.

Disclosing one’s TB status for the family is important to get family support and reminder which helps to adhere. In this study, 15.3% of the participants did not get family support. This is supported by a study done in Mekelle which stated a lack of caregivers and people who remind the TB patients to take their medications were associated with non-adherence [14]. A systematic review stated Social support, support from family were identified as extremely important for TB treatment adherence [20]. Patients usually did not disclose their status because of fear of discrimination [23]. So, disclosing one’s status is important to get different support which can help to adhere to treatment.

In this study, one of the reasons for non-adherence to anti-TB treatment was past anti-TB treatment history. This is in line with studies done in Northeast Ethiopia, Northern Ethiopia, and Gambella Regional State, Ethiopia stated Patients with previously treated cases have a high level of treatment failure, and patients who had lost to follow-up history could be reluctant and tend to interrupt their treatment again and be non-adherent [24–26].

Having no information about the expected adverse event was also another reason for non-adherence to anti-TB treatment. This is supported by a qualitative study done in India which patients who had inadequate information on the management of the expected adverse event or problems to be non-adherent [23]. This could be when participants had counseled and had information on the expected adverse event they expect the adverse event and know the measures to take when they experienced these effects which help to adhere.

The other reason for anti-TB nonadherence among TB patients was smoking cigarettes. This finding is supported by a study done in Nigeria which stated that cigarette smokers had poorer TB treatment than a non-smoker [27]. This might be due to Cigarette smoke is known to damage the lungs and suppresses the individual adaptive immune responses affecting patients’ response to TB treatment which could lead to non-adherence [7, 28]. In contrast, a study done in southern Ethiopia mentioned smoking has no association with non-adherence.

In this study, some factors which were associated in different studies were not found to be significant determinants of non-adherence. For example, distance from a health facility, transportation cost, relationship with a health worker, and proper counseling [15, 16, 25].

One limitation of this study was the level of non-adherence to anti-TB treatment was self-reported. One of the main limitations of self-reporting adherence measurement is lack of reliability. This can be explained by patient recall bias and other information biases, social desirability, or the interviewer’s skills and construction of the questions. A close relationship between the patient and the healthcare professional could also result in an underestimation of non-adherence rates. So, it has been strongly recommended to use them as a complement of another method like the direct methods, for example, measurement of drug or metabolites and ingestible sensors which are more accurate [29]. Another limitation was a cross-sectional study was conducted, it might suffer from temporal relationship establishment with some variables and might not provide much stronger evidence of causality.

## Conclusion**s**

The level of Non-adherence among TB patients was high. Information on the expected adverse event, having past anti-TB treatment history, disclose TB status to family, and smoking cigarettes were factors independently associated with non-adherence to anti-TB treatment.

Health care providers should counsel TB patients about anti-TB treatment adverse events and measures to be taken when patients face adverse events. They should also counsel their patients to disclose their TB status to family and the effect of smoking on the treatment outcome.

## Additional file

Additional file 1 Questionnaire for the study.

## Data Availability

The data used to support the findings of this study are not publicly available due to restrictions on the publication of human subjects’ data but these data can be available from the corresponding author upon reasonable request.

## Abbreviations

AMGH: Arba Minch General Hospital
AMU: Arba Minch University
HIV/AIDS: Human Immune Deficiency Virus/ Acquired Immune Deficiency Syndrome
MDR: Multi-Drug Resistant
PTB-SM + ve: Smear Positive Pulmonary Tuberculosis
PTB-SM –ve: Smear Negative Pulmonary Tuberculosis
RVI: Retroviral Infection
ART: Antiretroviral therapy
OIs: Opportunistic Infections
