# Supplementary material for: Level of and associated factors for non-adherence to anti-tuberculosis treatment among tuberculosis patients in Gamo Gofa zone, southern Ethiopia: cross-sectional study
Source: BMC Public Health. 2020 Nov 13;20:1705. doi: 10.1186/s12889-020-09827-7 (PMC7666453; doi:10.1186/s12889-020-09827-7)
Supplement: Supplementary file 1 — Questionnaire for the study. [file 12889_2020_9827_MOESM1_ESM.docx]

# Additional files

## Questionnaire for the study

**1. Socio demographic characteristics**

| S. No | Question | Alternative choice of response | Skip to | Code |
| --- | --- | --- | --- | --- |
| 1. | Residence | 1.Urban  2. Rural |  |  |
| 2. | Sex of Respondent | 1. Male 2. Female |  |  |
| 3. | Age of respondent  (Years) | 1. 15 - 24 3. 35 - 44  2. 25 – 34 4. ≥ |  |  |
| 4. | What is your marital Status? | 1. Married 2. Single 3. Divorced 4. Widowed |  |  |
| 5. | What is your Religion? | 1. Orthodox  2. Muslim  3. Protestant  4. Catholic  5. Other (Specify) |  |  |
| 6. | What is your ethnicity? | 1. Gamo 2. Gofa 3. Konso 4. Oromo 5. Other (Specify) |  |  |
| 7. | Family income monthly | 1. < 500 2. ≥500 |  |  |
| 8. | What is your current educational status? | 1. Unable to read & write 2. Read & write 3. Grade 1to 8 4. Grade 9 to 12 5. College and above |  |  |
| 9. | What is your occupation? | 1. Daily laborer 2. Government employer 3. Farmer 4. Private employer 5. Driver 6. House wife 7. Other (specify) |  |  |

1. **Healthcare system and other related characteristics**

| 10. | How do you come here? Transport | 1. On foot 2. Public transport 3. Private car |  |  |
| --- | --- | --- | --- | --- |
| 11. | How long does it take for you to reach to the health facility? | 1. ≤30 minutes  2. >30 minutes |  |  |
| 12. | How long do you wait before you get served? | 1. ≤30 minutes  2. >30 minutes |  |  |
| 13. | How is the availability of drugs? | 1. Always available  2. Not always available |  |  |
| 14. | Have you been given counseling and education? | 1. Received 2. Not received |  |  |
| 15. | Have you been given Health education on adherence and side effects of the treatment? | 1. Given 2. Not given |  |  |
| 16. | How is health workers approach? | 1. Very friendly 2. Friendly 3. Unfriendly |  |  |

**3. Patient Related characteristics**

| S. No | Question | Alternative choice of response | Skip to | Code |
| --- | --- | --- | --- | --- |
| 17. | Have you missed dose of anti TB in the past 4 days? | 1. Yes 2. No |  |  |
| 18. | Have you missed dose of anti TB in the past month? | 1. ≥ 3 times 2. < 3 times |  |  |
| 19. | Reason for missing | 1. Forgetfulness 2. Travelling to other places 3. Death of family members 4. Far distance 5. Side effect of the tx 6. Too many drugs to take |  |  |
| 20. | Family member or friend to remind patient to take RX | 1. Yes 2. No |  |  |
| 21. | Knowledge about TB  A, Etiology B. Mode of transmission C. Symptom  D. Treatment duration E. Side effect of treatment | 1. Knowledgeable   (if ≥ 3)   1. Non-knowledgeable 2. (if <3) |  |  |
| 22. | Have tried other options as treatment like spiritual and holy water? | 1. Yes 2. No |  |  |
| 23. | TB status disclosure to the family | 1. Disclosed 2. Not disclosed |  |  |
| 24. | Felt discriminated by family/Community | 1. Discriminated 2. Non discriminated |  |  |
| 25. | Who is supporting you in your treatment? | 1. Health facility worker 2. A family member 3. No treatment supporter |  |  |
| 26. | Alcoholism | 1. Yes 2. No |  |  |
| 27. | Chewing chat | 1. Yes 2. No |  |  |
| 28. | Smoking | 1. Yes 2. No |  |  |

**4. Anti – TB Treatment and disease related characteristics**

| S. No | Question | Alternative choice of response | Skip to | Code |
| --- | --- | --- | --- | --- |
| 29. | Experience of side effects | 1.Yes  2. No |  |  |
| 30. | Side effect experienced | 1. Skin rash 2. Head ache and Dizziness 3. Yellow eyes 4. Vomiting 5. Urine discoloration |  |  |
| 31. | Symptom of tuberculosis during interview | 1. Yes 2. No |  |  |
| 32. | Type of TB | 1. PTB-SM +ve 2. PTB-SM –ve 3. EPTB |  |  |
| 33. | Treatment History | 1. New 2. Re-treatment |  |  |
| 34. | HIV screening status | 1. Screened 2. Not screened |  |  |
| 35. | HIV status | 1. Positive 2. Negative |  |  |
| 36. | ART status | 1. Started 2. Not started |  |  |
| 37. | OIs other than TB | 1. Yes 2. No |  |  |
